# Oral Health Status of Children With a History of Liver Transplantation

**DOI:** 10.1111/petr.70125

**Published:** 2025-06-29

**Authors:** Güler Burcu Senirkentli, Simge Polat, N. Sena Onder

**Affiliations:** ^1^ Department of Pediatric Dentistry, Faculty of Dentistry Baskent University Ankara Turkey

**Keywords:** chronic liver disease, dental age, dental caries

## Abstract

**Objectives:**

Chronic liver disease (CLD) in children imposes a significant health burden that impacts their development. This study aims to investigate how dental health outcomes change after liver transplantation in children, focusing particularly on the post‐transplant period, examining the effect of immunosuppressive therapy, dental and skeletal anomalies in this patient group, caries experience, and dental age‐related factors, and to develop targeted interventions for these problems.

**Methods:**

The prevalence of dental caries, the presence of odontogenic anomalies, the presence of jawbone anomalies, the need for periodontal treatment, the need for orthodontic treatment, and dental age were evaluated.

**Results:**

Fifty children (aged 6–12 years) with CLD and fifty healthy controls participated in the study.

The analysis revealed no significant differences between the groups in terms of dental caries prevalence, presence of odontogenic anomalies, or jawbone anomalies. However, a statistically significant difference was found in dental age. While the analysis revealed no significant differences in dental caries prevalence between the groups, it is important to consider the possible moderating role of regular dental follow‐ups after transplantation.

**Conclusions:**

These findings offer valuable insights for dental professionals and pediatricians managing children with CLD. Future research with a larger sample size could further explore the association between CLD and dental age while investigating the potential influence of factors such as disease severity and medication use on oral health.

## Graphical abstract

This study examines the oral health of children who have undergone liver transplantation, highlighting dental development delays despite similar caries rates compared to their healthy peers. Findings emphasize the need for integrated dental‐medical care, focusing on dental age assessment and early intervention to optimize oral health outcomes in this vulnerable population.

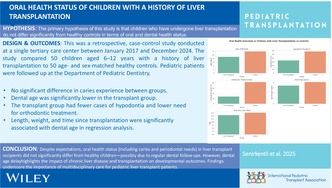

## 1 Introduction

Chronic liver disease (CLD) in children is characterized by progressive and often irreversible damage to the liver, leading to significant morbidity and mortality [1]. This damage, characterized by fibrosis and potentially progressing to cirrhosis, significantly impairs liver function [1, 2]. The complications of CLD can include such as cholestasis, portal hypertension, fluid build‐up, bleeding from enlarged veins (variceal bleeding), compromised protein production, blood clotting issues (coagulopathy), hepatic encephalopathy, and disruptions in kidney and lung function (hepatorenal and hepatopulmonary syndromes) are major concerns [2]. Nutritional deficiencies, metabolic imbalances, and weakened immunity further complicate the situation, increasing the risk of malnutrition and infections [3]. Oral health is also affected, with reported manifestations such as enamel defects, reduced saliva production, and lesions on the oral mucosa [3]. Recent reviews further confirm the prevalence of enamel defects and mucosal lesions in children undergoing liver transplantation, highlighting Candida infections and pigmentation changes as common findings [4]. A 2023 scoping review also noted the frequent occurrence of enamel hypoplasia, pigmentation, and candidiasis in children pre‐ and post‐transplantation [5].

Despite the growing recognition of CLD as a pediatric health issue, the underlying pathophysiological mechanisms and optimal management strategies remain incompletely understood. Previous research focused primarily on the hepatic manifestations of the disease, with limited attention paid to extrahepatic complications and quality of life outcomes. Moreover, there is a paucity of studies investigating the oral health implications of CLD in children. To address these knowledge gaps and inform the development of comprehensive care plans, there is a critical need for further research exploring the multisystemic effects of CLD in children, including its impact on oral health [6]. This study aligns with the latest international guidelines from the American Academy of Pediatric Dentistry (AAPD) and European Society of Pediatric Gastroenterology, Hepatology and Nutrition (ESPGHAN), which emphasize the importance of multidisciplinary management of pediatric liver transplant patients, including dental monitoring [7, 8].

The etiology of CLD in children varies by age group. In infants, persistent cholestasis often underlies the disease, with causes ranging from infections and metabolic disorders to genetic conditions, structural abnormalities, and autoimmune diseases [1]. Biliary atresia and genetic‐metabolic diseases are the most common causes, while chronic viral hepatitis and autoimmune disorders are more common in older children [3, 9]. In a significant number of cases, however, a definitive cause remains elusive [3, 9].

Liver transplantation offers a life‐saving solution for children with end‐stage CLD, dramatically improving their survival rates and quality of life [10]. However, to prevent organ rejection and promote growth, immunosuppressive medications become a lifelong necessity. Although advancements such as tacrolimus, which is a commonly used immunosuppressant drug to prevent organ rejection after transplantation, have reduced chronic rejection rates, these drugs have their own drawbacks, including a negative impact on oral and dental health [11]. In our study, oral health data were collected during the post‐transplant follow‐up period and thus reflect post‐transplantation status rather than preoperative oral health conditions.

In children with liver transplantation, there are particular susceptibilities to certain oral problems. Green discoloration of the teeth and gums due to pretransplantation bilirubin levels is a common finding [12]. Additionally, cyclosporine, a frequently used immunosuppressant, can lead to enlarged and inflamed gums (gingivitis) [12]. Other factors, such as poor oral hygiene, dental hypoplasia (underdeveloped teeth), and increased cavities, are also more prevalent in this population [13, 14]. The null hypothesis of the present investigation posits that no statistically significant difference exists between the groups.

This study seeks to address the critical gap in knowledge regarding the oral health implications of children of liver transplantation by investigating the prevalence of dental and skeletal anomalies, as well as DMFT/dmft (decayed, missing and filled teeth) scores and dental age, in this patient population. Unlike previous research primarily focused on the hepatic manifestations of chronic liver disease, this study specifically examines the oral health outcomes of children who have undergone liver transplantation. By comparing these findings to a healthy control group and assessing dental age, we aim to elucidate the unique dental challenges faced by this patient population and contribute to the development of targeted oral healthcare interventions.

## 2 Materials and Methods

### 2.1 Study Design and Setting

The current study was designed as a retrospective cohort study on pediatric patients with a preliminary diagnosis of liver damage and follow‐up at Baskent University Hospital between January 2017 and December 2024. The study was carried out at Baskent University, Department of Pedodontics. Ethical approval for the study was received from the Baskent University Medicine and Health Sciences Research Board (D‐KA23/22), and the study was conducted in accordance with the Declaration of Helsinki. Oral health data were collected during the post‐transplant follow‐up period and reflect the post‐transplant status rather than the pre‐operative oral health status.

As all patients underwent oral health optimization, including clinical and radiographic examinations and dental treatments, before transplantation surgery, the study findings reflect the characteristics of chronic liver disease. Patients who had passed away or for whom complete clinical and radiological data, such as panoramic radiographs, could not be obtained were excluded from the study. Patients with liver damage who met the inclusion criteria were included in this study, and healthy individuals were included as the control group (Table 2). The study group was compared to a control group comprising children attending expressed by: Baskent University Department of Pedodontics who were matched for age (6 and 12) and sex. The inclusion criteria for the subjects in the control group were children in good health (ASA I) with or without a history of dental care. The control group consisted of children attending routine pediatric dental follow‐up visits, and not selected based on any known systemic conditions.

The dental structure (caries, odontogenic anomalies, etc.) and clinical and radiographic data of the jaw bones of all patients were evaluated retrospectively. In addition, the demographic characteristics of the patients (age, sex, height, weight and body mass index), type and dose of immunosuppressive agent used, need for periodontal treatment, and need for orthodontic treatment were recorded. In children of liver transplantation, the need for periodontal treatment was evaluated based on panoramic films, and if the value was outside the numerical values considered healthy, based on the AAPD guideline and reference to the cementoenamel border, the presence of alveolar bone loss was assessed [7]. Patients who are planned to have liver transplantation are provided with complete oral hygiene before transplantation and are seen in frequent follow‐ups afterwards and detailed treatment notes are written. In our study, the orthodontic treatment needs of the patients were evaluated by examining both panoramic and intraoral examination information. All the data were compared statistically between patients with liver damage and healthy patients.

### 2.2 Panoramic Radiographs

Panoramic radiographs (Morita Veraviewepocs 2DCP, J. Morita INC., USA) were used for radiological evaluation.

### 2.3 Dental Age Determination

The dental age determination was carried out after the panoramic radiographs were converted to TIFF format in grayscale using the InterPacs (13.2.19401.1661, Health Solutions Industry Trade Limited Company, Turkey) program and saved as photographs. To evaluate the stages of tooth development more clearly, only mandibular molar teeth were evaluated with Demirjian's method [15].

### 2.4 Dental Caries and Its Incidence

Dental caries prevalence was assessed using the dmft index for primary teeth and the DMFT index for permanent teeth. DMFT/dmft represent the total number of decayed, missing (due to caries), and filled teeth. Caries incidence was determined as the percentage of participants with a DMFT/dmft score greater than zero. To accurately calculate DMFT/dmft, a thorough clinical examination of each participant's oral cavity was conducted by a calibrated examiner using standard dental instruments under adequate lighting conditions.

### 2.5 Odontogenic Anomalies

The presence and type of odontogenic anomalies (number, size, shape, structural anomalies, and presence of enamel defects in the teeth), caries, pulp chamber (pulp stones), and bone status were evaluated. Third molars (wisdom teeth) were not assessed as the prevalence of hypodontia in any of the groups.

### 2.6 Statistical Analysis

Analysis of the data was performed using SPSS version 22 (IBM SPSS Statistics for Windows, Armonk, NY: IBM Corp.). The mean ± standard deviation and median (minimum‐maximum) were used as descriptive statistics for quantitative variables, and the number of patients (percentage) was used for qualitative variables. The normality test of the distribution was performed with the Kolmogorov–Smirnov test. In terms of the quantitative variable, whether there was a difference between the categories of the qualitative variable with two categories was examined using Student's *t* test if normal distribution assumptions were met and the Mann–Whitney *U* test if not. When the relationship between two qualitative variables was examined, the chi‐square test and Fisher's exact test were used. The level of statistical significance was accepted as *p* < 0.05.

## 3 Results

### 3.1 Study Population

**FIGURE 1:**
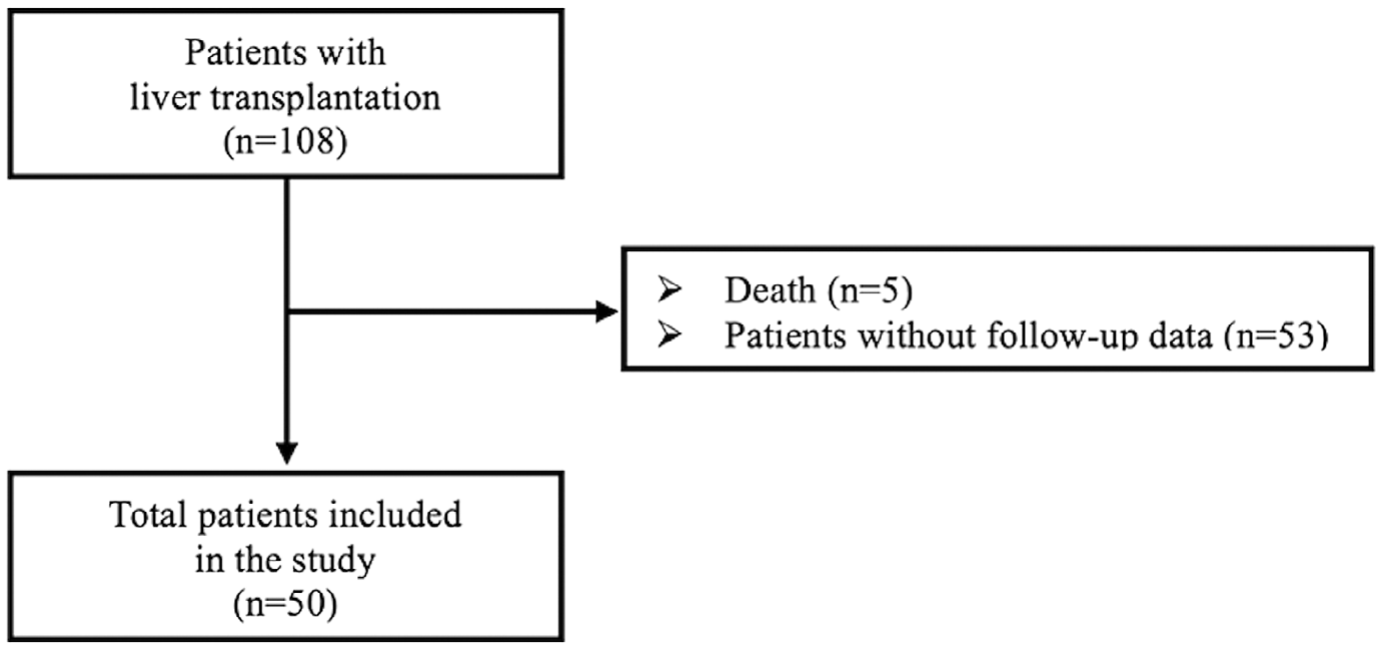
Flow chart of patients included in the study.

A total of 100 children participated in this study; 50 (31 boys and 19 girls) children aged 6–12 years who had a history of CLD comprised the study group, and 50 healthy children (27 boys and 23 girls) aged 6–12 years served as controls (Figure 1). The primary diagnoses of the children with CLD, listed in Table 1, were as follows: biliary atresia, neonatal hepatitis, familial cholestasis, and Alagille syndrome. The other causes included cirrhosis in 18 patients each, hepatoblastoma, primary hyperoxaluria, Crigler–Najjar syndrome and glycogen storage disease type I (Tables 1 and 2).

**TABLE 1:** Primary diagnoses of children with chronic liver disease.

Primary diagnosis	Patients (*n* = 50)
Biliary atresia [*n* (%)]	15 (30%)
Neonatal hepatitis [*n* (%)]	4 (8%)
Familial cholestasis [*n* (%)]	9 (18%)
Alagille syndrome [*n* (%)]	4 (8%)
Crigler–Najjar syndrome [*n* (%)]	2 (4%)
Primary hyperoxaluria [*n* (%)]	3 (6%)
Hepatoblastoma [*n* (%)]	2 (4%)
Cirrhosis [*n* (%)]	8 (16%)
Glycogen storage disease type I [*n* (%)]	3 (6%)

**TABLE 2:** Comorbidities and immunosuppressive agents used in patients with liver transplantation.

Comorbidity [*n* (%)]	
DM	1 (2%)
HT	1 (2%)
Thyroid diseases	0 (0%)
Heart diseases	2 (4%)
Gastrointestinal diseases	1 (2%)
Epilepsy	0 (0%)
Metabolic diseases and syndromes	10 (20%)
Immunosuppressive agents	
Mycophenolate mofetil	
*n* (%)	44 (88%)
Mean daily dose (mg)	450 (120–1000)
Tacrolimus	
*n* (%)	50 (100%)
Mean daily dose (mg)	1 (0.5–4)
Glucocorticoid	
*n* (%)	43 (86%)
Mean daily dose (mg)	80 (15–500)

### 3.2 Dental Caries, Anomalies and Need for Treatment Outcomes

There was no difference in the mean DMFT/dmft between the study and control groups (*p* = 0.451 and *p* = 0.075, respectively) (Table 3). Moreover, there was a significant difference in the presence of hypodontia (*p* = 0.016; *p* < 0.05) and the need for orthodontic treatment (*p* = 0.002; *p* < 0.05) (Table 3), but there was no significant difference in terms of jawbone anomalies or the need for periodontal treatment (*p* > 0.05) (Table 3).

**TABLE 3:** Comparison of basic demographics, physical examination, radiographic imaging findings, and dental health parameters between children of liver transplantation and healthy control group.

	Liver transplant patients (*n* = 50)	Control group (*n* = 50)	*p*
Age (years)	6.56 ± 2.37	7.15 ± 2.01	0.247
Dental age (years)	6.89 ± 2.57	8.21 ± 1.94	0.013*
Δ age (years)	0.32 ± 1.31	1.05 ± 0.66	0.010*
Gender [*n* (%)]			0.544
Female	19 (38%)	23 (46%)	
Male	31 (62%)	27 (54%)	
Length (m)	0.91 ± 0.24	1.25 ± 0.14	< 0.001*
Weight (kg)	12.5 (5.6–26)	25 (15–58)	< 0.001*
BMI (kg/m^2^)	15.75 ± 3.45	17.72 ± 2.96	0.003*
Time after transplantation (years)	5 (0.1–11)	—	N/A
Dental caries [*n* (%)]			0.356
Yes	40 (80%)	35 (70%)	
No	10 (20%)	15 (30%)	
dmft (*n*)	8 (1–16)	8 (0–20)	0.451
DMFt (*n*)	1 (1–4)	4 (0–5)	0.075
Odontogenic anomalies [*n* (%)]			
Hypodontia	17 (34%)	29 (58%)	0.016*
Supernumerary tooth	0 (0%)	2 (4%)	0.495
Persistent tooth	1 (2%)	2 (4%)	1.000
Size and shape anomalies			
Microdontia	1 (2%)	0 (0%)	1.000
Macrodontia	0 (0%)	0 (0%)	N/A
Deformed tubercle	0 (0%)	2 (4%)	0.495
Enamel pearl	0 (0%)	0 (0%)	N/A
Taurodontism	0 (0%)	0 (0%)	N/A
Reduced incisal edges of incisors	3 (6%)	2 (4%)	1.000
Pulp stones [*n* (%)]	0 (0%)	0 (0%)	N/A
Jaw bone anomalies [*n* (%)]	0 (0%)	0 (0%)	N/A
Need for periodontal treatment [*n* (%)]	3 (6%)	3 (6%)	1.000
Need for orthodontic treatment [*n* (%)]	7 (14%)	21 (42%)	0.002*

*Note:* *p* < 0.05 was considered statistically significant and significant *p* values are marked with * in the table.

Abbreviations: N/A, not applicable; Δ age, dental age—chronological age.

### 3.3 Dental and Physiological Age Outcomes

**FIGURE 2:**
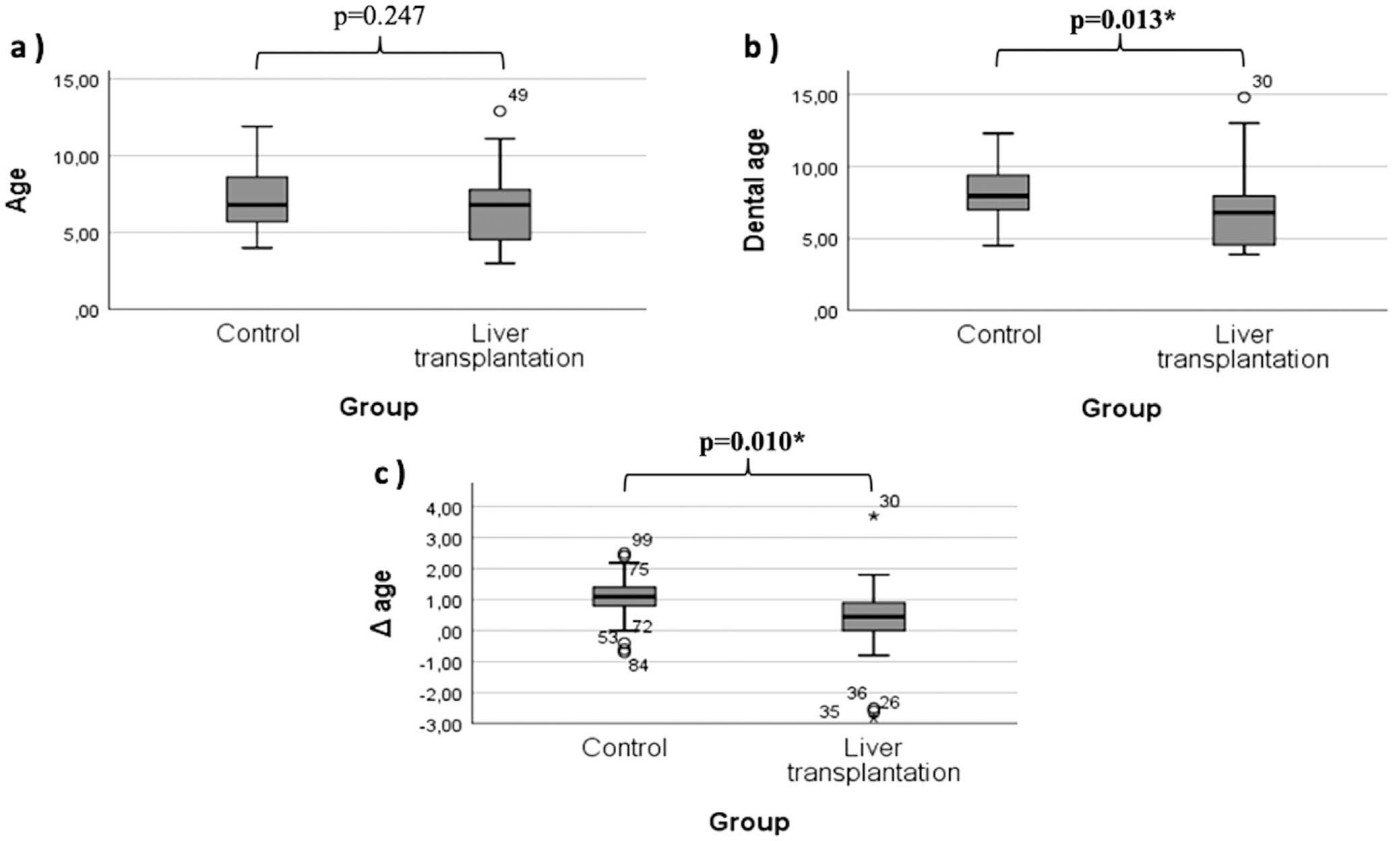
Box‐plot graph of age (a), dental age (b), and delta age (c) of liver transplantation and control groups.

When we evaluated the groups within themselves, no significant difference between dental age and physiological age was found. In addition, when dental age was evaluated between groups, it was lower in individuals with chronic liver disease (*p* = 0.013) (Table 3, Figure 2). When body mass indices were compared between the groups, the body mass index of the control group was greater (*p* = 0.003) (Table 3).

Table 4 shows the linear regression analysis of the independent variables affecting dental age. In the model, only length, weight and time after transplantation positively and significantly affected dental age (*p* = 0.001, *p* = 0.002 and p = 0.001, respectively). In addition, in the correlation analysis, dental age was moderately correlated with all three variables (*r* = 0.418, *r* = 0.475 and *r* = 0.429, respectively).

**TABLE 4:** Evaluation of the model created from independent variables affecting dental age in children of liver transplantation using linear regression analysis.

Model	*B*	Standardized coefficients beta	95% confidence interval		Correlations		*p*
			Lower	Upper	Zero‐order	Partial	
Gender	0.176	0.041	−1.228	1.581	0.127	0.075	0.790
Length (m)	8.670	0.970	4.014	13.325	0.418	0.745	0.001*
Weight (kg)	0.269	0.897	0.118	0.419	0.475	0.731	0.002*
BMI (kg/m^2^)	−0.012	−0.015	−0.298	0.274	0.145	−0.025	0.928
Comorbidity	−0.210	−0.046	−1.791	1.370	−0.001	−0.079	0.778
Time after transplantation (years)	0.514	0.799	0.252	0.777	0.429	0.762	0.001*
Mean daily dose (mg)							
Mycophenolate mofetil	0.002	0.235	−0.001	0.005	0.039	0.316	0.251
Tacrolimus	0.512	0.151	−0.990	2.014	0.454	0.200	0.474
Glucocorticoid	−0.006	−0.347	−0.013	0.002	0.289	−0.414	0.125

*Note:* *p* < 0.05 was consired statistically significant and significant *p* values are marked with * in the table.

## 4 Discussion

Chronic liver disease has a more significant impact on children's overall health, oral health, and quality of life than on those of adults [16]. Fortunately, liver transplantation has become a widely accepted treatment for children with CLD in recent years, significantly improving their long‐term survival rates [17]. This means that dental professionals are increasingly likely to encounter these patients in their clinics. However, research on the oral health of children with CLD, particularly those with end‐stage liver disease or those who have received liver transplants, is limited. Our understanding of the oral health complications in children with liver diseases is primarily based on case reports, indicating a need for more extensive research [18]. In this study, no difference was found in mean DMFT/dmft between children with liver transplantation and control groups. One possible explanation lies in our hospital's referral process, where doctors routinely refer patients to dentists for regular check‐ups and necessary procedures. There is a published study in which the DMFT/dmft values are greater than those in healthy patients [19], as well as studies in which there is no statistically significant difference, similar to the results of our investigation [4, 20]. However, larger cohort studies and reviews have begun to emerge, including works such as the 2019 study on Kuwaiti children and a recent 2024 pediatric transplant cohort review [16, 21]. Thus, the null hypothesis was accepted.

Within‐group analysis revealed no significant difference between dental and physiological age. Furthermore, children with CLD exhibited a lower dental age compared to their chronological age, which supports existing knowledge about how CLD can hinder bone development [22]. A recent study by Sarici et al. showed that the bone and mineral metabolism of liver transplant recipients are adversely affected after liver transplantation [23]. Although dental age and bone age have undergone different biological processes, a study by Saraç et al. on Turkish children showed a positive correlation between chronological age, dental age, and skeletal age [24]. In another study examined the relationship between dental calcification and skeletal maturation stages, all correlations between dental age and skeletal age were statistically significant [25]. The findings of these studies are consistent with the results of our study.

BMI analysis revealed a statistically significant difference between the groups, with the control group exhibiting a higher mean BMI. Although no research has directly investigated dental age in children with chronic liver disease (CLD), this condition is well documented to impair the development of bone tissue, which is another hard, mineralized tissue similar to teeth. Children with CLD frequently experience a range of bone development problems, including decreased bone mineral density (BMD), reduced bone mass, increased risk of fractures, and bone deformities. Furthermore, sarcopenia and reduced muscle mass, commonly observed in pediatric CLD, might contribute to lower BMI values in this group [26].

When the results were evaluated, dental age was positively and significantly influenced by the independent variables, which were length, weight, and time since transplantation. It has been concluded in the literature that early organ transplantation in children with growth retardation is optimal for restoring their growth potential, while delaying transplantation in older children hinders potential growth [27, 28]. Liver transplantation should be performed as soon as possible after the diagnosis of liver failure to minimize the degree of growth retardation in recipients prior to transplantation. Studies have shown that the most catch‐up growth is more likely to occur in younger children [29].

This study investigated the impact of chronic liver disease on the dental health of children who underwent transplantation. We compared the prevalence of dental caries, odontogenic anomalies, jawbone anomalies, the need for periodontal treatment, and the need for orthodontic treatment between children with CLD and healthy controls. Additionally, we evaluated the dental age in both groups. Interestingly, there were no significant differences in DMFT/dmft scores and the prevalence of jawbone anomalies between the groups. In the study conducted by Schmalz et al., it was observed that immunosuppressive drugs (tacrolimus, cyclosporine, mycophenolate, glucocorticoids) were not associated with the need for dental and periodontal treatment [30]. In this study, similar results were found to the results of our study. In addition, there was significant difference in presence of hypontia and the need for orthodontic treatment. In present study, the presence of hypodontia and the need for orthodontic treatment were found to be higher in the control group. When the results of our study were evaluated, the higher rate of hypodontia and orthodontic treatment requirements in the control group may have been due to the random selection of control group patients. In addition, it is possible that clinicians may adopt a more conservative approach in CLD and post‐transplant patients due to the presence of systemic comorbidities and increased risk of complications. The regular medical follow‐up associated with post‐transplant care may have contributed to improved oral hygiene and dental care adherence among CLD patients. Furthermore, the discrepancy in the need for orthodontic treatment between the groups may also reflect differences in access to dental care. Post‐transplant patients often receive comprehensive healthcare, including dental care recommendations, as part of their follow‐up, which could potentially influence the observed differences in treatment needs. Future studies with larger cohorts and more detailed assessments of orthodontic treatment decisions, oral hygiene practices, and dental care adherence are warranted to further elucidate these findings. Previous studies have evaluated oral health and dental manifestations such as DMFT, plaque scores, gingival overgrowth, and hypoplasia in patients with chronic liver disease, but there is a gap in the literature regarding the presence of dental anomalies and the need for orthodontic treatment [14, 16, 20]. This suggests that with proper dental care and regular dentist visits, children with CLD may not experience greater rates of tooth decay or developmental problems with their teeth and jaws than do healthy children. However, our findings did reveal a statistically significant difference in dental age, with children with CLD exhibiting a lower dental age compared to their chronological age. Moreover, our results support conclusions from recent systematic reviews summarizing oral health risks in pediatric transplant populations [31, 32], highlighting delayed dental development. This finding aligns with existing research on how CLD can hinder bone development. A potential limitation of the current study is that due to its retrospective design, we were not able to evaluate patient‐related factors (oral hygiene practices, dietary habits, dentist compliance). One potential explanation is that clinicians may adopt a more conservative approach in children with CLD due to systemic vulnerabilities, or that CLD patients, under regular medical follow‐up, may exhibit better oral hygiene habits and adherence to dental visits. Another limitation is the lack of detailed data on post‐transplant complications such as episodes of rejection, steroid use, infections, and hospitalizations, all of which could have an impact on oral and dental health. This aligns with findings from a 2017 investigation on post‐transplant oral health, which confirmed the role of immunosuppressants in soft tissue conditions, but not necessarily in caries development [33].

## 5 Conclusion

The results of this study offer valuable insights for both dental professionals and pediatricians caring for children with CLD. Although dental caries may not be a major concern, regular dental check‐ups and preventive care remain crucial for this population. Furthermore, the potential for delayed dental development in children with CLD necessitates close monitoring. Collaboration between dental and medical professionals can ensure optimal oral health outcomes for these patients. Future research with a larger sample size could further explore the association between CLD and dental age while also investigating the potential influence of factors such as disease severity and medication use on oral health.

## Author Contributions

**Güler Burcu Senirkentli:** conceptualization, formal analysis, methodology, supervision, software, project administration, validation, visualization, writing – original draft, and writing – review and editing. **N. Sena Onder:** data curation, methodology, resources, software, and writing – review and editing. **Simge Polat:** formal analysis, investigation, methodology, resources, software, and writing – review and editing.

## Conflicts of Interest

The authors declare no conflicts of interest.

## Data Availability

The data that support the findings of this study are available from the corresponding author upon reasonable request.
